# The potential prognostic value of connexin 26 and 46 expression in neoadjuvant-treated breast cancer

**DOI:** 10.1186/1471-2407-13-50

**Published:** 2013-02-02

**Authors:** Ivett Teleki, Tibor Krenacs, Marcell A Szasz, Janina Kulka, Barna Wichmann, Cornelia Leo, Barbel Papassotiropoulos, Cosima Riemenschnitter, Holger Moch, Zsuzsanna Varga

**Affiliations:** 1 1st Department of Pathology & Experimental Cancer Research, Semmelweis University, Budapest, Hungary; 2 MTA-SE Tumour Progression Research Group, Budapest, Hungary; 3 2nd Department of Pathology, Semmelweis University, Budapest, Hungary; 4 2nd Department of Internal Medicine, Semmelweis University, Budapest, Hungary; 5 Division of Gynecology, University Hospital Zurich, Zurich, Switzerland; 6 Breast Cancer Center Seefeld, Zurich, Switzerland; 7 Institute of Surgical Pathology, University Hospital Zurich, Zurich, Switzerland

**Keywords:** Breast cancer, Connexin, Gap junction, Preoperative chemotherapy, Prognosis

## Abstract

**Background:**

Several classification systems are available to assess pathological response to neoadjuvant chemotherapy in breast cancer, but reliable biomarkers to predict the efficiency of primary systemic therapy (PST) are still missing. Deregulation of gap junction channel forming connexins (Cx) has been implicated in carcinogenesis and tumour progression through loss of cell cycle control. In this study we correlated Cx expression and cell proliferation with disease survival and pathological response to neoadjuvant chemotherapy in breast cancers using existing classification systems.

**Methods:**

The expression of Cx26, Cx32, Cx43, Cx46 and Ki67 was evaluated in 96 breast cancer patients prior to and after neoadjuvant chemotherapy using duplicate cores in tissue microarrays (TMA). Cx plaques of <1μm were detected with multilayer, multichannel fluorescence digital microscopy. Current classifications to assess residual tumour burden after primary systemic therapy included the EWGBSP, CPS-EG, Miller-Payne, Sataloff and NSABP systems.

**Results:**

In our cohort dominated by hormone receptor (ER/PR) positive and HER2 negative cases, only the CPS-EG classification showed prognostic relevance: cases with scores 1–2 had significantly better overall survival (p=0.015) than cases with scores 3–5. Pre-chemotherapy Cx43 expression correlated positively with hormone receptor status both before and after chemotherapy and had a negative correlation with HER2 expression pre-chemotherapy. There was a positive correlation between Cx32 and HER2 expression pre-chemotherapy and between Cx32 and Ki67 expression post-chemotherapy. A negative correlation was found between post-chemotherapy Cx46 and Ki67 expression. Decreased post-chemotherapy Cx26 expression (<5%) statistically correlated with better overall survival (p=0.011). Moderate or higher Cx46 expression (>20%) pre- and post-chemotherapy correlated with significantly better survival in the intermediate prognostic subgroups of EWGBSP TR2b (p_pre-chemo_=0.006; Sataloff TB (p_pre-chemo_=0.005; p_post-chemo_=0.029) and in Miller-Payne G3 (p_pre-chemo_=0.002; p_post-chemo_=0.012) classifications. Pre-chemotherapy, Cx46 expression was the only marker that correlated with overall survival within these subgroups.

**Conclusion:**

Our results suggest that Cx46 and Cx26 expression in breast cancer may improve the assessment of pathological response and refine intermediate prognostic subgroups of residual tumour classifications used after neoadjuvant chemotherapy.

## Background

Despite of mammographic screening, early diagnosis and available targeted therapy, breast cancer is still one of the most frequent cause of tumour related death of women in the western world [1]. Molecular subtyping and related new therapeutic approaches require diagnostic screening for at least the hormone receptors (ER, PR) and HER2 overexpression/gene amplification as predictive and prognostic markers [2]. To date several classifications have been developed to assess pathologic response to neoadjuvant chemotherapy. Most systems are based on the amount of residual tumour in the breast and the axilla [3-7]. The recently described CPS-EG score combines clinical and pathological stage with nuclear grade and hormone receptor status [8,9]. However, reliable biomarkers to prognosticate response to primary systemic therapy (PST) are still missing.

The gap junction forming connexins (Cx) mediate direct cell-cell communication and their dysfunction can contribute to carcinogenesis and tumour progression in a wide range of neoplasias including breast cancer [10]. Connexins form gap junction channels that allow the regulated transport of <1.5kDa molecules, including secondary messengers (Ca^2+^, cAMP, IP3), metabolites (ATP, NAD^+^, small peptides and nucleotides) between adjacent cells [11] to coordinate functions within cell compartments [12]. They are abundant in all human solid tissues and more than one isotype - of the 21 cloned human isotypes - are found in most cell types [13]. Connexins play crucial roles in cell homeostasis including of regulation of cell growth, proliferation and apoptosis, either as gap junctions, hemichannels or through protein-protein interactions [14,15]. Therefore, functional Cx can be localized both to the cell membrane and the cytoplasm [16]. In normal mammary epithelium, Cx43 has been found in the myoepithelial cells and Cx26 in the luminal epithelium [17].

Connexins and gap junctions have been linked to carcinogenesis through aberrant expression and functions [18]. They show tumour stage dependent expression and may play opposing roles during breast cancer progression [19]. Connexin expression is usually down-regulated upon malignant transformation, but it can also mediate tumour cell endothelial interactions during tumour diapedesis [20]. Cx43 and Cx26 can be involved in tumour suppression in early stage breast cancer, but these connexins and Cx32 may also support metastatic tumour colonization by upregulation in the lymph nodes [21-23]. Moreover, new connexin isotypes such as Cx46 can appear in breast cancer, which may assist MCF-7 breast cancer cells in adapting to hypoxia [24].

The aim of this study was to correlate connexin expression and cell proliferation with clinicopathological parameters (stage, ER, PR and HER2) and prognosis in breast cancer patients treated with PST. Of the 15 of 21 connexin isotypes (Cx23, Cx26, Cx30, Cx30.2, Cx30.3, Cx31, Cx31.1, Cx32, Cx36, Cx37, Cx43, Cx45, Cx46, Cx50 and Cx62) tested in a pilot study Cx26, Cx32, Cx43 and Cx46 were detected reliably and widespread in breast cancers. These isotypes were screened together with Ki67 expression prior to- (pre-chemo) and after neoadjuvant chemotherapy (post-chemo) using whole-slide immunofluorescence digital microscopy in 96 breast cancers included in tissue microarrays (TMA). Results were correlated with subgroups of the current classifications including the CPS-EG score (Clinical Pathological Stage combined with Estrogen receptor status and Grade by M.D. Anderson Cancer Center, Texas), NSABP (National Surgical Adjuvant Breast and Bowel Project), Miller-Payne ‘G’, Sataloff ‘T’ and EWGBSP ‘TR’ (European Working Group for Breast Screening Pathology) systems [3-9]. Our results suggest the potential prognostic value of Cx detection in neoadjuvant treated breast cancer. Cases with reduced Cx26 expression post-chemotherapy and those with moderate to high Cx46 expression both pre- and post-chemotherapy showed significantly improved survival rates particularly in the intermediate subgroups of current classification systems.

## Methods

### Patient cohort

96 patients with breast cancer, diagnosed either in core biopsies or fine needle aspiration biopsy (FNAB) and then treated with neoadjuvant chemotherapy were selected consecutively between 1998 and 2009 from the archives of the Institute of Surgical Pathology, University Hospital Zurich, Switzerland. Chemotherapy regimens included Docetaxel 75 mg/m^2^; Epirubicin 90 mg/m^2^; Cyclophosphamide 500 mg/m^2^; Doxorubicin 50 mg/m^2^; Vinorelbine 30 mg/m^2^; Fluorouracil 500 mg/m^2^; and also Trastuzumab (250 mg/m^2^). The exact preoperative chemotherapy schedules were available in 73 patients (Docetaxel/Epirubicin n=24, Epirubicin/Cyclophosphamide /Fluorouracil n=23, Docetaxel/Epirubicin/Cyclophosphamide n=14, Docetaxel/Trastuzumab n=7, Vinorelbine/Trastuzumab n=5). Chemotherapy schedules were administered in 2 to 6 cycles.

Formalin-fixed, paraffin-embedded (FFPE) tumour blocks from preoperative core biopsies and from the corresponding postoperative tissues were available in 64 patients. Core biopsies prior to neoadjuvant chemotherapy without surgical specimens after chemotherapy were available in 17 patients. Surgical specimens following the chemotherapy without previous core biopsies (diagnosed with FNAB) were available in 15 patients. Clinicopathological and follow-up data (2 to 10 years) on 96 patients could be retrieved from the pathological and clinical files as summarized in Table 1. Histologically or cytologically 75 tumours (78%) were diagnosed as invasive ductal carcinoma, 18 (19%) as invasive lobular carcinoma, 2 cases were metaplastic squamous cell carcinomas (2%) and one case was a small cell carcinoma. Patient’s age ranged from 30 to 74 years (mean age: 52 years). Eight of 96 patients (9%) had multifocal tumours on imaging. Histological grading according to the modified Bloom-Richardson score could be done in 81 cases: 38 cases (40%) were poorly differentiated (grade 3) carcinomas, 42 (44%) moderately differentiated (grade 2), one case (1%) was well differentiated (grade 1), 15 cases (15%) had only small amount of tumour tissue insufficient for correct grading on the core [25].

**Table 1:** Clinicopathological features of breast cancer patients studied

**n=96**	**Prior to chemotherapy**		**After chemotherapy**	
**Tumour size**	1,5-13 cm		0,3-14 cm	
	cT1	1 (1%)	ypT0	6 (6%)
	-a		ypT1	20 (21%)
	-b		-a	2
	-c	1	-b	11
	cT2	25 (26%)	-c	7
	cT3	22 (23%)	ypT2	31 (32%)
	cT4	41 (43%)	ypT3	24 (25%)
	-b	19	ypT4	7 (7%)
	-d	22	-b	6
	NA	7 (7%)	-d	1
			No surgery	8 (9%)
**Lymph node status**	cN0	10 (11%)	pN0	25 (26%)
	cN1	62 (64%)	pN1	27 (28%)
	cN2		pN2	12 (12%)
	cN3	3 (3%)	pN3	14 (15%)
			No surgery	8 (9%)
	NA	21 (22%)	NA	10 (10%)
**ER status**	Positive	68 (71%)	Positive	59 (62%)
	Negative	25 (26%)	Negative	16 (17%)
	NA	3 (3%)	NA	21 (21%)
			ypT0, no surgery	
**PR status**	Positive	59 (62%)	Positive	46 (48%)
	Negative	34 (35%)	Negative	29 (31%)
	NA	3 (3%)	NA	21 (21%)
			ypT0, no surgery	
**HER2 status**	Positive	28 (29%)	Positive	18 (19%)
	Negative	65 (68%)	Negative	57 (60%)
	NA	3 (3%)	NA	21 (21%)
			ypT0, no surgery	
**Ki67 status**	0	2 (2%)	0	22 (28%)
	1 (0-1%)	12 (15%)	1	21 (27%)
	2 (1-5%)	19 (23%)	2	11 (14%)
	3 (5-10%)	13 (16%)	3	3 (4%)
	4 (10-15%)	6 (7%)	4	1 (1%)
	5 (15-20%)	4 (5%)	5	6 (8%)
	6 (20-33%)	2 (2%)	6	1 (1%)
	7 (33-50%)	4 (5%)	7	2 (3%)
	8 (50-66%)	1 (1%)	8	0 (0%)
	9 (66-80%)	3 (4%)	9	3 (4%)
	10 (80-100%)	1 (1%)	10	2 (3%)
	NA	14 (17%)	NA	7 (9%)
	∑	64+17 (100%)	∑	64+15 (100%)

NA: Accurate data are not available.

Mastectomy was performed in 60 patients (62%) and segmentectomy in 28 patients (29%). Breast surgery was completed with axillary dissection in all but 5 of these patients. In 8 patients (9%) either no surgery was performed or we had no records in our files. Fifteen of 96 patients (16%) had multifocal tumours at the time of surgery. Tumour cellularity was estimated as the percentage of residual tumour cells distributed in the tumour bed area [6]. The histological subtypes were identical to those of the preoperative biopsies. The frequency of ER/PR and HER2 positive cases in our cohort roughly reflected that of the general breast cancer population.

The study and the construction of TMA was approved by the Ethical Committee of the Canton Zurich (KEK-ZH NR: 2009–0065) and also by the Internal Review Board of the Institute of Surgical Pathology.

### Detection of hormone receptors and HER2

Estrogen receptor (ER, clone 6F11) and progesterone receptor (PR, clone 1A6) expression was determined using the iVIEW DAB detection kit in Ventana Benchmark (all from Ventana, Basel, Switzerland) immunostainer following heat induced epitope retrieval in CC1 solution. Cases of >1% nuclear positive tumour cells were considered as positive [25].

HER2 status was defined according to the initial and the modified ASCO criteria using immunohistochemistry and/or fluorescence *in situ* hybridization (FISH) (1998–2004 IHC and FISH, 2004–2009 only FISH) [26].

Between 1998-2004 the Pathway™ HER2 (clone CB11) FDA approved kit (Ventana) was used for automated immunostaining as described above. Cases with tumour cells of >10% strong and complete membrane staining were considered 3+; and those with >10% moderate, but complete membrane staining as 2+ requiring additional FISH testing. During the 1998–2009 period *HER2* gene amplification was tested using the dual colour FISH kit of PathVision (Vysis, Abbott AG, Baar, Switzerland) according to the manufacturer’s protocol.

### Tissue microarray construction

Hematoxylin-eosin (H&E) stained sections of all FFPE tumours were re-evaluated by one pathologist (Z.V.) for suitability for TMA, which were prepared as described earlier containing duplicate cores from each patient’s samples [27,28]. Core biopsies from 81 patients prior to chemotherapy (64 matched surgical and core biopsy; and 17 core biopsy only) and tumour samples from 79 patients after chemotherapy (64 paired as above and 15 surgical) were arrayed into two TMA blocks.

### Immunofluorescence detection of connexins

TMA slides of 4 μm thick were dewaxed in xylene and rehydrated through ethanol series. Antigen unmasking was done in a 0.1 M Tris 0.01 M EDTA buffer (pH 9.0) using an electric pressure cooker (Avair, Biofa, Veszprem, Hungary) for 20 min at ~105 °C followed by 10 sec digestion in 0,25% Gibco trypsin phenol red (1:50; Life Technologies, Carlsbad, CA, Ref: 25050–014). After a protein blocking step for 20 min the slides were incubated overnight using rabbit anti-mouse Cx26 (1:4000, AB8143, Millipore, Billerica, MA) or anti-human Cx32 (1:30, HPA010663, Sigma-Aldrich, St Luis, MO), Cx43 (1:100, #3512, Cell Signaling, Beverly, MA) or Cx46 (1:100, SAB13005557, Sigma-Aldrich) antibodies. All animal specific connexin antibodies used highly cross-react with the relevant human connexins [29]. TMA slides were simultaneously also stained for Ki-67 protein using the mouse anti-human Mib-1 antibody (1:2 ready-to-use, IR626, Dako, Glostrup, Denmark).

Fluorochrome-labelled secondary antibodies, Alexa Fluor 546 (red) goat anti-rabbit IgG (A11035) and Alexa Fluor 488 (green) goat anti-mouse IgG (A11001) were applied in 1:200 for 90 min. Finally, Hoechst (1:1000, B2883, blue) was used for nuclear staining (all from Invitrogen-Life technologies, Eugene, OR), for 60 sec. All incubations were done in a humid chamber at room temperature and slides were washed between the steps using 0.1 M TBS (Tris-buffered saline) pH 7.4 for 2x5 min. Immunostained slides were digitalized using Pannoramic Scan (3DHISTECH Ltd., Budapest, Hungary). The multilayer, multichannel fluorescence digitalization of TMA slides resulted in permanent fluorescence samples and allowed accurate analysis of connexin plaques of frequently <1μm size within the whole section thickness without the risk of false negativity due to fading or lack of focus depth resolution.

### Scoring of connexin expression and cell proliferation

Immunoreactions for Cx-s were evaluated by two independent assessors using a 4-scale scoring system with the TMA module software [30] (3DHISTECH) by considering the frequency of positively stained cells as follows: score 0: <5%; score 1: 5-20%; score 2: 20-60%; score 3: >60%). Positive signals of all assessed connexin subtypes were localized in the plasma membrane in the normal breast epithelial cells. In breast cancer samples, positive reaction was mainly seen in the cytoplasm of the invasive tumour cells. Both of these signal types were considered upon scoring. Stromal, smooth muscle and endothelial cells, adipocytes, skin, keratinocytes and normal mammary epithelium served as endogenous positive controls. Ki-67 reaction was assessed on a linear 0–10 scale (score 0: 0, 1: 0-1%, 2: 1-5%, 3: 5-10%, 4: 10-15%, 5:15-20%, 6: 20-33%, 7: 33-50%, 8: 50-66%, 9: 66-80%, 10: 80-100%) considering the frequency of positive tumour cells (Table 1). In case of tumour heterogeneity the higher score was considered for statistics.

### Assessing tumour response after neoadjuvant chemotherapy using histopathological and clinical classification systems

Five current pathological classification systems were applied retrospectively to assess pathologic response after neoadjuvant chemotherapy. Four of these systems NSABP, Miller-Payne Grade, Sataloff T and EWGBSPTR analyse the extent of residual tumour tissue (none, *in situ* or invasive). These could be applied retrospectively in 89 cases. The CPS-EG score classification combines clinical and pathological stages with nuclear grading and hormone receptor expression, which could be used in 55 cases. Details of pathological response and definition of classification systems are summarized in Table 2.

**Table 2:** Definition of classification systems and distribution of the enrolled cases

**Classification system**		**Cases**	**Pathological response**			
**NSABP**	pCR	5	No histological evidence of invasive tumour cells			
	pINV	84	Histological evidence of invasive disease of any extent			
**Miller-Payne**	G1	10	Some alteration to individual malignant cells but no reduction in overall numbers as compared with the pre-treatment biopsy			
	G2	20	A minor loss of invasive tumour cells but overall cellularity still high (<30%)			
	G3	40	A moderate reduction of in tumour cells up to an estimated 90% loss (30-90%)			
	G4	14	A marked disappearance of invasive tumour cells such that only small clusters of widely dispersed cells could be detected (>90%)			
	G5	5	No invasive tumours, i.e., only in situ disease or tumour stroma remained			
**Sataloff**	TA	20	Total or near total therapeutic effect			
	TB	30	Subjectively greater than 50 % therapeutic effect but less total and near total			
	TC	29	Less than 50% therapeutic effect, but effect event			
	TD	10	No therapeutic effect			
**EWGBSP**	TR1a	4	No residual tumour			
	TR1b	1	No residual invasive tumour but presence of residual in situ carcinoma			
	TR2a	14	Minimal residual invasive tumour (<10%)			
	TR2b	31	Therapeutic effect with residual invasive tumour (10-50%)			
	TR2c	29	Therapeutic effect but >50% residual invasive tumour			
	TR3	10	No pathologic response			
**CPS-EG**	0	0	**Point assignments**			
	1	2	Clinical stage	Pathologic stage	Tumour marker	
	2	19	I; IIA = 0	0; I = 0	ER negative =1	G3^4^ =1
	3	17	IIB; IIIA = 1	IIA/B; IIIA/B = 1		
	4	12	IIIB; IIIC = 2	IIIC = 2		
	5	5				
	6	0				

^1^*NSABP*: National Surgical Adjuvant Breast and Bowel Project

^2^*EWGBSP*: European Working Group for Breast Screening Pathology

^3^*CPS-EG*: Clinical Pathological Stage combined with Estrogen receptor status and Grade by M.D. Anderson Center (MDACC)

^4^G3: grade

### Statistical analysis

SPSS 15.0 software was used for statistical comparisons (SPSS, Inc., Chicago, IL, USA). Categorical data were analysed using chi-square test. Spearman rank correlation was used to correlate connexin scores and clinicopathological parameters (stage and grade, hormone receptor status, HER2 status). Overall survival was calculated using Kaplan-Meier method with assessment of statistical significance by log-rank test. For multivariate analysis Cox-regression analysis with 95% confidence intervals was performed by including Cx26, Cx46, hormone receptor status, HER2 status, pre-chemo tumour size, proliferation, pathological response, stage and grade which did not correlate directly with each other. Results were considered statistically significant at p-values of <0.05. Bonferroni correction was not applied.

## Results

### Connexin expression before and after neoadjuvant chemotherapy

**Figure 1:**
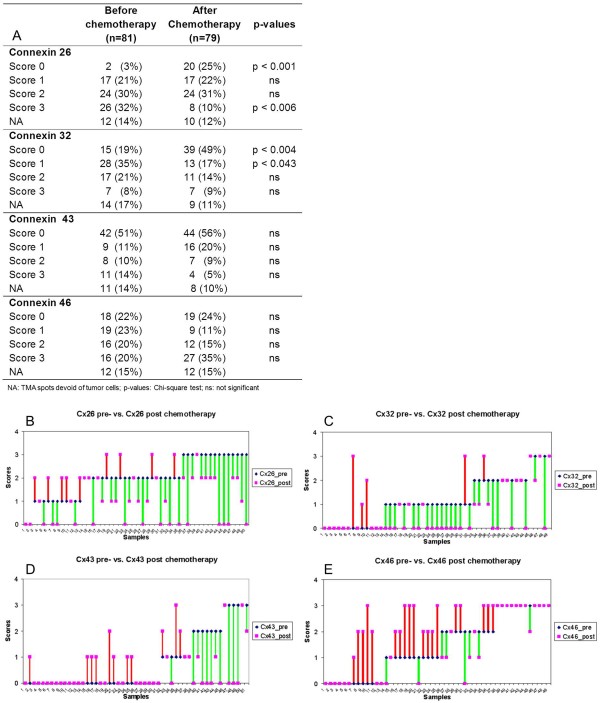
**Expression of connexin isotypes in breast cancers before and after neoadjuvant chemotherapy (A).** Dynamic changes in connexin isotype expression in the 50 (in Cx43, 51) matched breast cancer samples upon treatment (**B-E**). Cx26 (**B**) and Cx32 (**C**) levels mostly decreased, Cx43 (**D**) levels revealed the least change, while Cx46 (**E**) levels were mainly increased. Green lines represent decrease, red lines highlight increase. Violet symbols alone represent no change in expression.

**Figure 2:**
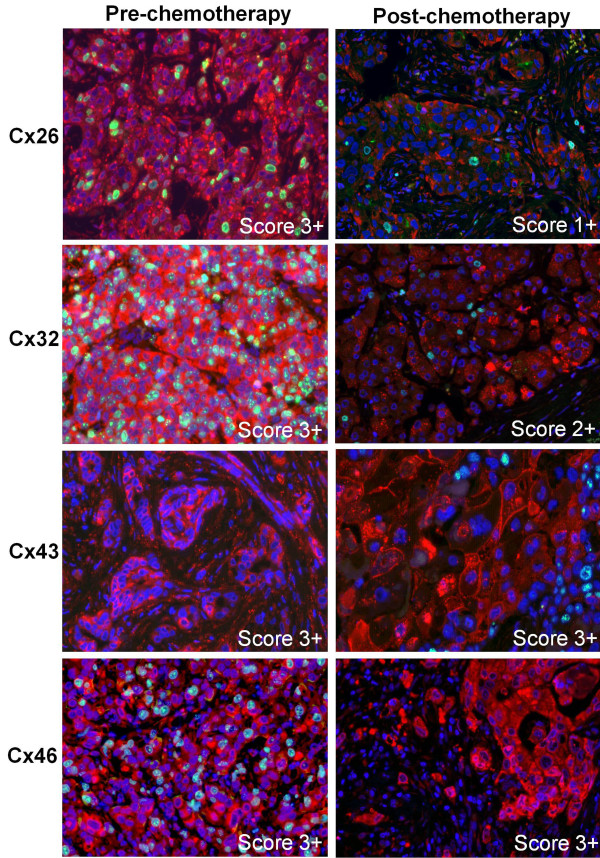
**Immunofluorescence detection of Cx26, Cx32, Cx43, Cx46 (Alexa 546, red) and Ki67 (Alexa 488, green) in invasive ductal breast cancers prior to (pre-) and after (post-) neoadjuvant chemotherapy.** Cell nuclei were stained using Hoescht (blue). Punctuate cell membrane associated and cytoplasmic Cx26 (1st row) and Cx32 (2^nd^ row) levels and the proliferating (green) tumour cell fractions were reduced after neoadjuvant therapy in cases originally scoring 3+. Cx43 (3^rd^ row) and Cx46 (4^th^ row) levels did not change after neoadjuvant chemotherapy in cases of high scores (3+). Pre-chemotherapy Cx43 and post-chemotherapy Cx46 samples are not stained for Ki67.

Connexin expression upon chemotherapy showed dynamic but differential changes related to the isotypes which is also revealed in details for individual cases in (Figure 1). Examples of related connexin immunofluorescence are seen in Figure 2. In the whole patient cohort, the number of Cx26 expressing tumours and the percentage of Cx26 positive tumour cells in individual tumours significantly decreased after chemotherapy. The number of cases scoring 0 increased from 3% to 25% (p <0.001), while the number of cases scoring 3 decreased from 32% to 10% (p <0.006). The frequency of intermediate scores (1 and 2) did not change during therapy. Cx32 expression was significantly reduced after chemotherapy. The frequency of cases scoring 0 (negative) increased from 10% to 49% (p <0.004) while of those scoring 1 (weak positive) decreased from 35% to 17% (p <0.043). The frequency either of Cx43 or Cx46 positive cases did not differ significantly before and after chemotherapy, though, the number of 3+ Cx46 positive cases showed a tendency of increase post-chemotherapy. Of 64 matched samples 50 (for Cx43 51 cases) could be assessed reliably. Cx26 and Cx32 were mainly decreased, Cx43 showed the least change while Cx46 levels showed an increasing tendency upon chemotherapy **(**Figure 1B-E**).**

### Correlation of connexin expression with clinicopathological parameters before and after neoadjuvant chemotherapy

Correlations between connexin expression, cell proliferation and clinicopathological features are summarized in Table 3.

**Table 3:** Correlations of connexin and Ki67 proliferation marker expression with clinicipathological parameters

	**Cx43**		**Cx26**		**Cx32**		**Cx46**		**Ki67**	
	pre	post	pre	post	pre	post	pre	post	pre	post
ER pre	0.30	x	ns	x	ns	x	ns	x	−0.46	−0.35
ER post	0.36	ns	ns	ns	ns	ns	ns	ns	−0.40	−0.38
PR pre	0.34	x	ns	x	ns	x	ns	x	−0.45	x
PR post	0.33	ns	ns	ns	ns	ns	ns	ns	−0.52	ns
HER2 pre	−0.27	x	ns	x	0.31	x	ns	x	ns	x
HER2 post	ns	ns	ns	ns	ns	ns	ns	ns	ns	0.26
cT pre	0.29	x	ns	x	ns	x	ns	x	ns	x
cN pre	ns	x	ns	x	ns	x	ns	x	ns	x
pT post	ns	ns	ns	ns	−0.29	ns	ns	ns	ns	ns
pN post	ns	ns	ns	ns	ns	ns	0.39	0.32	ns	ns
grade pre	ns	x	ns	x	ns	x	ns	x	ns	ns
Ki67 pre	ns	x	ns	x	ns	x	−0.29	x	x	0.46
Ki67 post	ns	ns	ns	ns	ns	0.46	ns	ns	0.46	x

ns: not significant; x: not relevant; pre: prior to chemotherapy; post: after chemotherapy.

**Figure 3:**
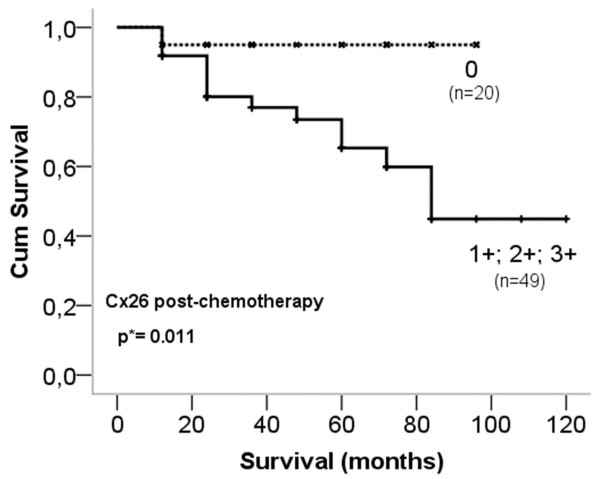
**Kaplan-Meier plot of overall survival based on Cx26 expression after chemotherapy.** Log-rank test reveals significant association (p=0.011) between reduced Cx26 expression (0; <5%) and improved disease outcome.

Cx26 expression in cases of <5% positive tumour cells (score 0) post-chemo statistically correlated with improved overall survival (p=0.011) compared to those of >5% Cx26 positive tumour cells (scores 1–3) (Figure 3). However, Cx26 expression did not show correlation with other clinicopathological parameters when using the Spearman rank (*ρ)* test. At the same time, Cx32 expression had a positive correlation with HER2 status both pre-chemo (*ρ=*0.31) and a negative correlation to pathological tumour stage (pT) (*ρ* =−0.29). Moreover, post-chemo Cx32 and Ki67 expression showed a positive correlation (*ρ*=0.46). Pre-chemo Cx43 expression positively correlated with ER and PR expression both pre- and post-chemo and with the clinical tumour stage (cT) (*ρ*=0.29-0.36) and had a negative correlation with HER2 status pre-chemo (*ρ*=−0.27). Cx46 expression pre- and post-chemo had a positive statistical correlation with nodal status (pN) (*ρ*=0.39 and 0.32) and pre-chemo Cx46 and Ki67 expression negatively correlated with each other (*ρ*=−0.29). A negative correlation was found between ER and Ki67 expression both pre- and post-chemo (ρ=−0.35- -0.46) and between PR and Ki67 expression pre-chemo (ρ=−0.45; -0.52). Furthermore, Ki67 expression had a positive correlation with HER2 status both post-chemo (ρ=0.26) and a positive correlation was detected between pre-and post-chemo Ki67 scores (ρ=0.46).

### Correlation between the defined clinicopathological response groups and overall survival

**Figure 4:**
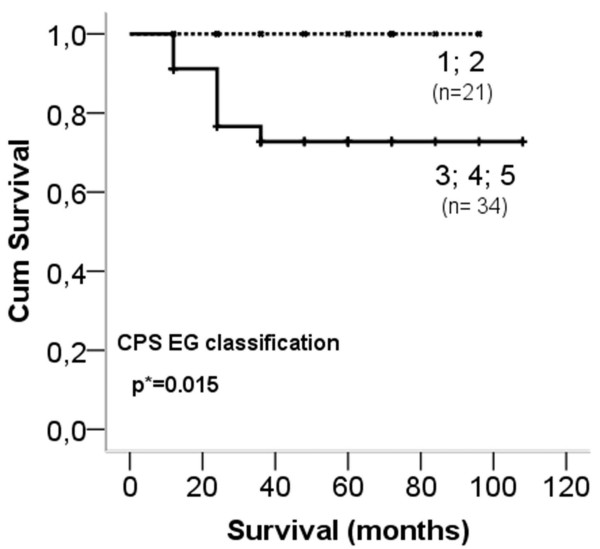
**Kaplan-Meier plot of overall survival based on the CPS-EG classification.** Cases scoring 1–2 show significantly improved overall survival (p=0.015) compared to those scoring 3–5.

Primary breast cancer cases were classified according NSABP, Miller-Payne, Sataloff, EWGBSP, CPS-EG systems. Only the CPS-EG classification showed correlation with overall survival: cases with scores 1 and 2 had significantly better survival rate (p=0.015) than those scoring 3, 4 and 5 (Figure 4).

### Overlap among subgroups of the classification systems

**Figure 5:**
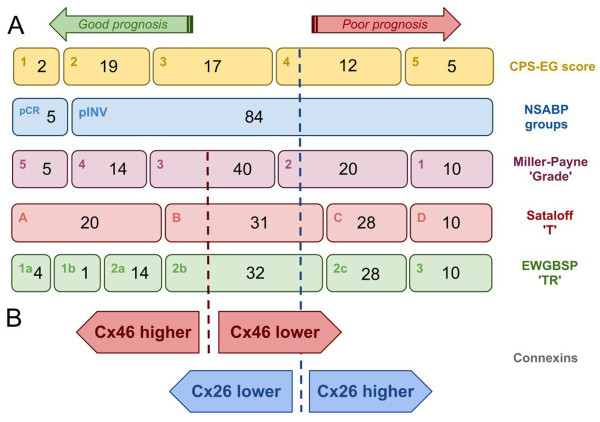
**Distribution of the studied patient cohort within current classifications and the power of Cx26 and Cx46 expression to discriminate good and poor prognostic groups within the equivocal categories of NSABP, Miller-Payne G, Sataloff T, EWGBSP TR and MDAAC CPS-EG systems that are used for assessing response to neoadjuvant chemotherapy (A).** Both high Cx46 levels and low Cx26 levels correlate with good prognosis (**B**).

We also tested how subgroups of the classification systems overlap in our patient cohort (Figure 5A). In general, only few cases fell into the subgroups with edge categories such as EWGBSP TR1a-b and TR3; CPS-EG 1 and 5; Sataloff TA and TD; or Miller-Payne GI and G5. These included the particularly responsive and the barely/non-responsive tumours which highly overlapped among classifications. The rest of cases were heterogeneously sorted into the intermediate categories including EWGBSP TR2a-TR2c; CPS-EG 2-4; Sataloff TB and TC; and the Miller-Payne G2-G4. The NSABP is a binary system without intermediate subgroups based on the presence or lack of residual tumours. Five cases fell into the pCR group of NSABP, the rest (84 cases) were assigned to the pINV group.

### Prognostic potential of connexin expression on residual tumour classifications

We analysed Cx26, Cx32, Cx43 and Cx46 expression within each classification subgroup in correlation with overall survival. Due to the Gaussian distribution of cases within the used classifications and the high agreement either within the good or the bad prognostic subgroups at the edge categories we focused on the intermediate subgroups.

**Figure 6:**
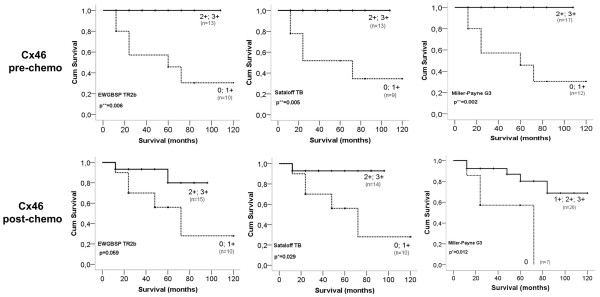
**Kaplan-Meier plot of overall survival based on Cx46 expression pre- and post- chemotherapy according to the morphological response rate assessed using the EWGBSP TR2b, Sataloff TB and Miller-Payne G3 classifications.** Elevated Cx46 expression both pre- and post-chemotherapy is associated with significantly better (p=0.002-0.05) overall survival in all of these categories.

Cases with 2+ and 3+ pre-chemo Cx46 expression (>20%) had significantly better survival rates in EWGBSP TR2b (p=0.006), Sataloff TB (0.005) and Miller-Payne G3 (p=0.002) subgroups, than those scoring 0 or 1+ (<20%) for Cx46. This correlation was almost the same for post-chemo Cx46 expression. In the Sataloff TB group the tumours with 2+ and 3+ Cx46 expression (>20%) had significantly better survival rates (p=0.029) than those scoring 0 and 1+ (<20%). In the Miller-Payne G3 category the tumours with 1-3+ Cx46 expression (>5%) showed significantly better survival (p=0.012) than those scoring 0 (<5%). In the EWGBSP TR2b group the tumours with 2+ and 3+ Cx46 scores showed nearly significant (p=0.059) better survival compared to cases scoring 0 and 1+ (Figure 6).

Cases with <5% Cx26 positive cells (score 0) had significantly better survival rates in NSABP pINV tumours than those with >5% (score 1+, 2+, 3+) Cx26 positive cells (p=0.013). Intermediate categories in the other subgroups also showed the same tendency but without statistical significance including EWGBSP TR2b (p=0.08), Sataloff TB (p=0.092), Miller-Payne ‘G2’ (p=0.053) and CPS-EG 4 (p=0.06).

### Prognostic potential of Cx26 and Cx46 in the whole patient cohort and in the subgroups

Using Kaplan-Meier estimates supported by log-rank test the expression of post-chemo Cx26, ER and PR status, pre- and post-chemo Cx46 as well as the CPS-EG classification showed statistical correlation with overall survival. Those features which were not correlated with each other among these when using the chi-square test were involved in multivariate Cox-regression analysis (Table 4). HER2 status, proliferation, grade, stage, pre-chemo tumour size, pathological response (tumour cellularity) did not correlate with overall survival, therefore, they were omitted from further analysis.

**Table 4:** Multivariate Cox-regression analysis of connexin expression, hormone receptors and CPS-EG classification in breast cancer patient groups

**Patient group**	**Parameters**	**P-value**	**HR**	**95% CI**	
				**Lower**	**Upper**
whole cohort	Cx26_post_	0.303	0.450	0.098	2.055
	ER_post_	0.050	2,739	0.999	7.504
	Cx26_post_	0.157	0.134	0.008	2.173
	PR_post_	0.027	6.509	1.241	34.140
	CPS EG	0.967	0.000	0.000	1.381E26
EWGBSP TR2b	Cx46_post_	0.790	4.400	0.844	22.950
	PR_post_	0.115	3.809	0.722	20.111
Sataloff TB	Cx46_post_	0.226	3.782	0.439	32.600
	PR_post_	0.244	2.791	0.496	15.711
Miller-Payne G3	Cx46_post_	0.104	3.322	0.782	14.105
	PR_post_	0.075	4.400	0.861	22.481

post: after chemotherapy; *HR*: hazard ratio; *CI*: confidence intervals.

In the whole patient cohort, cases with hormone receptor positivity after chemotherapy had a significantly improved overall survival (p_ER_=0.012, p_PR_=0.002). Post-chemo PR and ER showed a better independent prognostic value according to the multivariate analysis than post-chemo Cx26 levels and CPS-EG which latter two were statistically not significant (Table 4).

In the intermediate prognostic categories (EWGBSP TR2b, Sataloff TB and Miller-Payne G3) none of the tested parameters correlated with overall survival pre-chemo except Cx46_pre_, which thus proved to be the only potential prognostic factor. Post-chemo, only PR positive cases had a significantly better overall survival in the EWGBSP TR2b (p=0.03), Sataloff TB (p=0.05) and Miller-Payne G3 (p=0.006) subgroups. However, none of these markers proved to be significant independent prognostic factors in multivariate Cox-regression analysis (Table 4).

## Discussion

Neoadjuvant chemotherapy is now considered as one of the standard options in the treatment of locally advanced and also for primary operable breast cancers [5-8]. Morphological classification systems have been established to assess the pathological response and prognosis after systemic primary therapy. These consider pathological features such as the percentage of residual tumour cells, the presence or absence of *in situ* component with or without considering nodal status [3,5-8]. Reliable biomarkers to assess response to primary systemic therapy are not yet available. Connexins may function as gap junctions or hemichannels in the cell membranes or through intra-cytoplasmic protein interactions and play fundamental roles in cell homeostasis including cell cycle control [11,16]. In this study we analysed the expression of four connexin isotypes Cx26, Cx32, Cx43 and Cx46, and Ki67 for cell proliferation prior to and after neoadjuvant chemotherapy in breast cancer in correlation with clinicopathological parameters, overall survival and pathological response based on current classification systems.

Our main results suggest that increased Cx46 positivity either pre- or post-chemotherapy and reduced Cx26 expression post-chemotherapy may separate prognostically more favourable subgroups within the intermediate categories of the classifications including Miller-Payne G2-3, Sataloff TB, EWGBSP TR2b and CPS-EG 4 (Figure 5B). In addition, pre-chemotherapy only Cx46 expression appeared to correlate with overall survival. Others have also observed that biomarkers such as PR, HER2, grade, or tumour size did not necessarily correlate with prognosis either before or after chemotherapy [31]. In agreement with those who showed elevated Cx26 expression to contribute to carcinogenesis in pancreatic and prostate carcinoma [32,33] we also found a significantly reduced Cx26 expression after chemotherapy in association with better overall survival in breast cancer (p=0.011). Moreover, decreased Cx26 expression after chemotherapy could be more promising for prognosis than classical features such as HER2 expression, grade, Ki67 index according to our results. Interestingly, loss of Cx26 expression has also been implicated in reduced survival in primary gastric and colorectal carcinomas, suggesting an organ specific biological significance of Cx26 [34,35].

The role of connexins in breast cancer development, progression and metastatic growth has been the subject of only few previous studies all focusing on primary breast cancers [21-23,36]. The transitional loss of Cx43 has been reported where re-expression of Cx43 might sensitize breast cancer cell lines for chemotherapeutic agents [36]. Primary breast cancers exhibiting elevated expression of Cx26, Cx32 and Cx43 have been found more often in node positive than node-negative breast cancers [21,22]. Although, Cx46 has been implicated in protecting MCF-7 breast cancer cells from hypoxia, apart from our study, there has been no data revealed on the prognostic role of Cx46 in breast cancer [24]. Increased Cx26 and Cx43 levels have also been associated with breast cancer metastasis [22]. However, the prognostic and predictive role of connexins in relation to neoadjuvant chemotherapy has not been investigated before.

Gap junctions are thought to inhibit cell cycle though most findings relate to Cx43 [19]. Forced Cx43 expression can block G1/S phase transition or delay G2/M transition through increasing p21^waf1^ and reducing Cdk2 levels [37]. Also, Cx43 expression and gap junction coupling can be reduced through phosphorylation by Cdk1/cyclinB complex at G2/M transition [19]. Therefore, Cx expression may dynamically change during cell cycle. In breast cancer cell lines low levels of Cx26 at G1/S phase, increasing levels during late S/G2 phase transition and down-regulation in M phase was observed [38].

The correlations we have revealed between Cx expression and hormone receptor, HER2 or Ki67 levels suggest that connexin isotypes are differentially involved in the regulation of breast cancer cell functions. In concordance with published data we found a positive correlation between Cx43 expression and hormone receptor levels both before and after treatment in primary breast cancer [39]. However, either Cx43 or Cx26 protein can be elevated again in the lymph node metastasis of invasive breast cancer [22]. We also found a positive correlation between Cx32 and HER2 expression and between Cx32 and Ki67 expression; and a negative correlation between Cx43 and HER2 levels and between Cx46 and Ki67 expression. As expected, Ki67 expression showed a negative correlation with hormone receptor expression and a positive correlation with HER2 levels. These results may imply potential tumour suppressive functions for Cx43 and Cx46 in primary breast cancer as opposed to Cx26 or Cx32 expression which may be tumour protective as well. The positive correlation between Cx46 levels and nodal status may be explained by the lack of correlation between nodal status and overall survival in our cohort and that the majority of our cases were ER positive.

In this study, reduced Cx26 and Cx32 levels and elevated Cx46 expression upon chemotherapy may also reflect the efficiency of chemotherapy. The exact role of connexins in carcinogenesis and metastasis formation is controversial or more probably context dependent.

Several previous studies addressed the changes of biomarker expression after primary systemic therapy in breast cancer [40-43]. Discordance in hormone receptor status has been reported to vary from 2.5% to ~50% [41-43]. The same has been found for HER2 status with altered immunophenotype found in up to 43% of cases especially when immunohistochemistry was the choice of assay [42,43]. Although not analysed in detail here, we found discordant ER/PR/HER2 status in only <10% of our studied patients after neoadjuvant chemotherapy.

The predictive role of hormone receptors and HER2 status in view of partial and complete pathological response in breast cancer has also been extensively investigated [44-49]. HER2 positive, ER negative breast cancers with poor histological differentiation without nodal involvement have achieved better complete pathological response compared to those with lymph node metastasis [44,46-50]. In our cohort, ER positive HER2 negative tumours dominated representing prognostically intermediate categories. Therefore, we did not correlate hormone and HER2 expression with partial and complete pathological response to neoadjuvant therapy.

We have classified clinicopathological parameters in breast cancers according to five current systems and could establish a significantly improved overall survival in cases scoring 1–2 against those scoring 3–5 only when using the CPS-EG classification. The lack of correlation with overall survival in the rest of classifications (NSABP, Miller-Payne, Sataloff and EWGBSP) is probably due to the fact that the majority of our cases fell into the prognostically intermediate categories. As we are aware, comprehensive assessment of therapy response using the major classifications as we tested here has not been done so far by others in breast cancer treated with neoadjuvant therapy.

## Conclusion

Although our data are limited by the small sample size, they support additional studies of Cx26 and Cx46 to further refine outcome prediction for the intermediate groups as defined by currently used classification systems of pathological response to neoadjuvant chemotherapy in breast cancer.

## Competing interests

The authors declare no conflict of interest concerning the content of this manuscript.

## Authors’ contribution

ZV initiated this study with TK. Clinicopathological data were retrieved by CR in co-operation with CL and BP.CR made the tissue microarray. HM helped in writing the manuscript. TK supervised immunomorphological studies performed by IT and compiled the paper together with ZV and IT. IT, MSZ and BW performed the statistics after scoring together with JK. JK also gave advice on the interpretation of the results. All authors read and approved the final manuscript.

## Pre-publication history

The pre-publication history for this paper can be accessed here:

http://www.biomedcentral.com/1471-2407/13/50/prepub
